# Investigating the effects of superplasticizer and recycled plastics on the compressive strength of cementitious composites using neural networks

**DOI:** 10.1016/j.heliyon.2023.e21798

**Published:** 2023-10-31

**Authors:** Jindarat Ekprasert, Natthagrittha Nakhonthong, Vanchai Sata, Poemwai Chainakun

**Affiliations:** a Department of Microbiology, Faculty of Science, Khon Kaen University, Khon Kaen, 40002, Thailand; b School of Physics, Institute of Science, Suranaree University of Technology, Nakhon Ratchasima, 30000, Thailand; c Sustainable Infrastructure Research and Development Center, Department of Civil Engineering, Faculty of Engineering, Khon Kaen University, Khon Kaen, 40002, Thailand; d Center of Excellence in High Energy Physics and Astrophysics, Suranaree University of Technology, Nakhon Ratchasima, 30000, Thailand

**Keywords:** Neural network, Cement, Superplasticizer, Plastic waste, Compressive strength

## Abstract

The potential application of neural network (NN) models to estimate the compressive strength (CS) of cementitious composites under a variety of experimental settings and cement mixes was investigated. The data were extensively collected from previous literature, and the bootstrap resampling tests were applied to estimate the statistics of the parameter correlations. We find that the NN model that involves the coarse and fine natural aggregates (CA and FA), superplasticizer (SP) and recycled plastics (RP) as the features can accurately predict the CS (*R*^2^ ∼ 0.9), without the need to specify the type of SP and the structure of RP in advance. The developed NN model holds promise for revealing the global dependency of CS on these parameters. It suggested that increasing 100 kg/m^3^ of CA could increase CS by ∼4 MPa, but the usage of CA more than 700 kg/m^3^ could negatively affect CS. How the CS varying with FA is apparently nonlinear. Within the optimum limit, adding 1 kg/m^3^ of SP could enhance the CS by ∼2 MPa. Contrarily, additional 1 kg/m^3^ of RP results in a decrease of ∼0.2 MPa of CS. The mixture-type independent models developed here would broaden our understanding of the global influential-sensitivity among these variables and help save cost and time in the industrial applications.

## 1 Introduction

Plastics are frequently employed worldwide because of their beneficial features including lightweight, high durability, ease of processability and affordable prices [1]. The annual manufacture and consumption of these materials have been dramatically increased, resulting in a growing output of plastic wastes of approximately 6300 metric tons per year [2]. To eliminate these plastic wastes, 9 % are reused, 12 % are incinerated and the other 79 % are landfilled and discharged to the environment without prior treatments [2]. Therefore, the utilization of plastic wastes as either coarse or fine aggregates in place of natural aggregates for producing construction materials has become of interest [3,4].

Incorporating plastic wastes into cementitious materials leads to better performances in lightweight properties, sound absorption and thermal insulation of the materials, while the compressive strength is deteriorated [[5], [6], [7]]. The loss of strength is mainly because of the hydrophobicity of the plastic surface and the weakness of the plastic particles, which reduces cohesiveness between plastic and cement paste [[8], [9], [10]]. In order to overcome this issue, superplasticizers and/or pozzolanic materials like fly ash, silica fume, and blast furnace slag were incorporated into the materials [7,11,12]. However, an increase in pozzolanic contents, such as in the case of silica fumes, might require the use of more superplasticizers [11]. The use of superplasticizers, an anionic surfactant, as an admixture in plastic-cement materials can help increase the workability, slump retention and durability of the composites while reducing the need for water to mix the binders [13,14]. Therefore, the incorporation of superplasticizer affects the required dosage of other mixtures to gain maximum strength for these materials.

Previous literature has shown that, at a specific water-to-binder ratio, the compressive strength of plastic-containing concrete slightly increased with lowering the dosage of superplasticizer. Additionally, an increasing fine aggregate content caused a reduction in compressive strength when a higher dosage of superplasticizer was applied [5]. Due to its correlative effects with binders to the compressive strength, superplasticizer becomes an inevitable factor to be considered when used to improve the mechanical properties of plastic-cement materials.

Many attempts have been made to create desirable properties of plastic-cement composites, but there is still limited research on predictive analysis of their compressive strength. One of the very few studies applied 4 differential equations to predict the compressive strength of polyethylene terephthalate (PET)-fly ash geopolymer and cement mortar using porosity values including 1.) an exponential relationship between compressive strength and porosity of porous concrete (Ryshkewitch's model) and 2.) of mortar (Balshin's model), 3.) a logarithmic relationship between compressive strength and porosity of cement mortar (Schiller's model), and 4.) Hasselman's model which describes the relationship between compressive strength and porosity of Portland cement paste [7]. Despite considerable accurate prediction in some data (*R*^2^ ∼ 0.8), they only experimented with one type of plastic (polyethylene terephthalate; PET), and only one factor (void) was taken for predicting compressive strength. It is, therefore, still interesting to see how other binders and admixtures affect the compressive strength of general plastic-cement materials.

A few studies tried to develop mathematical models to predict compressive strength based on the effects of more than one cement mix [15]. These models could not make an accurate prediction due to characteristic differences between plastic and natural aggregates. To date, none of the existing mathematical models or expressions can be universally applied to predict the compressive strength of the cementitious materials incorporated with plastic wastes. Instead, the applications of machine learning algorithms are more promising (e.g., Duan et al. [16], Deshpande et al. [17], Salih et al. [18], Bu et al. [19] and Faraj et al. [20]). Among various ML methods, recent studies have proven that the neural network (NN) could be applied to accurately predict the compressive strength of cement under various conditions such as for recycled aggregate concrete [19] and plastic-cement composites [18,20]. It was found that the NN algorithm could perform better than other previously-reported models. Among these studies, different NN algorithms such as a multilayer perceptron [19] and a feedforward network [20] were used, and the employed data were also different due to different experimental setups.

Here, we focused on the possibility of using NN models to estimate the compressive strength of cementitious composites in a wider range of cement mixtures and experimental conditions. The data (i.e., cement samples) were extensively gathered from past studies from 2009 to 2021, in which the most recent work at the time we generated this research was included. While previous studies suggested that the compressive strength could be accurately constrained using the NN technique [[16], [17], [18], [19], [20]], the accurate prediction was often specific to the experimental designs (e.g., types of materials and mix proportions). The variation of experimental designs of the data gathered here then becomes a challenge for testing the NN model. The ultimate goal is to develop a NN model that can still predict the compressive strength of cement independently from the experimental designs and, perhaps, can also provide optimum values for key mixtures for a specified compressive strength. The estimated statistics for the correlations between the compressive strength of cement and other variables were performed using the bootstrap resampling technique. This allows us to confirm whether the obtained parameter correlations, either in the linear or monotonic way, are certain regarding the large scatter of the samples due to differences in experimental conditions set in previous studies. Then, the downstream NN models were developed to predict the compressive strength of cementitious composites using the initial parameters designed in the preparatory phase. The aim was to use the developed NN models to reveal the effects of superplasticizers and recycled plastics on the compressive strength of cementitious composites. The models should also be general enough (e.g., mixture-type independent) that they would be particularly important towards use in laboratory scales or future industrial applications.

## 2 Materials and methods

**Fig. 1:**
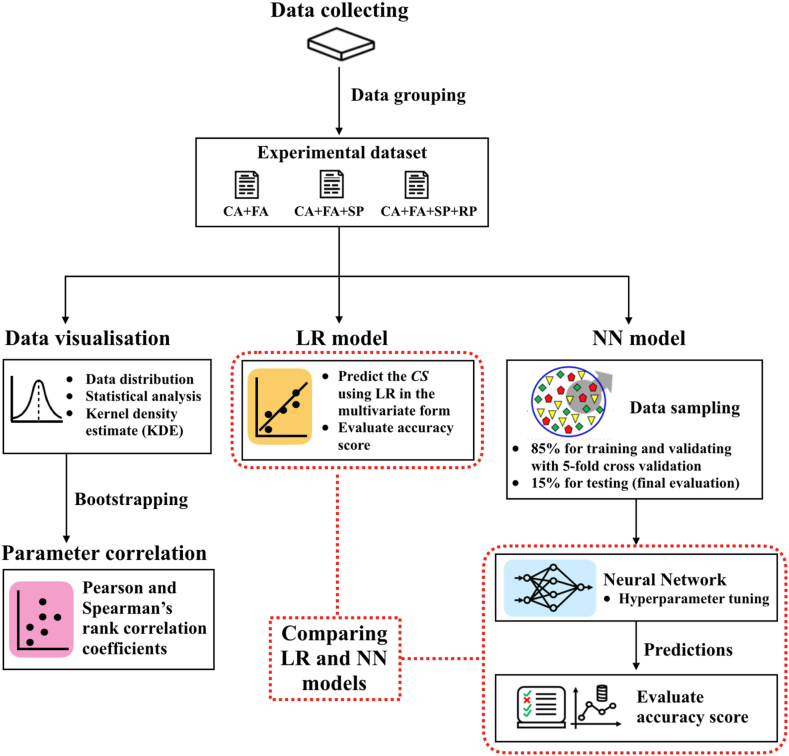
Flowchart of overall comprehensive research framework. See text for more details.

Firstly, we explained how the data was collected and grouped in different datasets in Section 2.1. Since the data were gathered from various sources, we first performed the data analysis to provide a comprehensive view of the data. The data visualization and statistical analysis used here were explained in Section 2.2. This also involved Pearson and Spearman's rank correlation coefficients, as well as the bootstrap resampling test that was used to analyze the robustness of the obtained correlations, as explained in Section 2.3. After that, the data were used for generating both linear regression and NN models to predict the unconfined compressive strength in order to compare their accuracy. The specific details of the linear regression and NN models were given in Sections 2.4 - 2.6. The flowchart representing the overall research framework is shown in Fig. 1.

### 2.1 Data samples

In this work, we extensively gathered past experimental data reported by Gravina et al. [15], Faraj et al. [20], Kumar & Baskar [21], Abdelatif et al. [22], and references therein. The data covered a variety of cementitious materials (e.g., mortar, concrete, and pozzolan-incorporated composites) and mixtures (e.g., high-density polyethylene (HDPE), polyvinylchloride (PVC)). More specific details of the data were also given in Appendix A. We extracted information including the 28-day compressive strength (CS) of the cube specimens, cement content (Cement), water-to-binder ratio (w/b), coarse aggregates (CA), fine aggregates (FA), superplasticizer (SP), and recycled plastics (RP). However, measuring correlation and developing ML can be much more difficult if the nature of the data within the same dataset is too different from each other. We then decreased the unnecessary complexity by grouping them into different datasets. For example, the data without SP and RP are grouped together. In this way, it is possible that we still find the common ground of the samples within the same dataset.

To capture a general picture of how each cementitious component affects the CS, the samples were grouped into 3 different datasets. We first began by sorting the cement samples that do not contain superplasticizers and plastic aggregates. These data were also gathered by Gravina et al. [15], but here we required further that the data included in our first dataset must contain full information on the Cement, w/b, CA, FA, and CS, and must be from the experiments without using SP and RP. Based on this cut, there are 452 cement samples left. This dataset is referred to as the (CA + FA) dataset and was provided as the supplementary data.

Next, the effects of the introduced superplasticizer and recycled plastics on the cement were investigated. These kinds of data were gathered before by researchers, such as Faraj et al. [20]. However, the second and third datasets here were produced, separately, by accumulating the data from earlier studies that have RP=0 and RP≠0, respectively, and were referred to as the (CA + FA + SP) and (CA + FA + SP + RP) datasets. For consistency, the data in these datasets must still have the full information of Cement, w/b, CA, FA, and CS. With these criteria, the (CA + FA + SP) and (CA + FA + SP + RP) datasets contain 64 and 60 samples in total, respectively, and both were also provided as supplementary data.

Note that the CS investigated here was measured at the material age of 28 days. Since one of our criteria was that the data must have full information specific to their dataset, there are no missing values at all in the datasets.

### 2.2 Visualizing data structures and correlations

The statistical analysis and data visualization were performed using the Orange data mining software [23]. The standard statistical values (e.g., minimum, maximum, mean, and dispersion) of the samples in each dataset were investigated individually. The distribution of main parameters of the cementitious components (Cement and w/b) when the CS were divided into three different groups (i.e., low, medium, and high CS) was calculated via Seaborn [24]. The layered kernel density estimate (KDE) was used to create the sample density distribution in order to reveal the visual differences between the data with different CS. We then statistically evaluated the correlations between the model variables using Pearson and Spearman's rank correlation coefficients, *r*_*P*_ and *r*_*S*_, that explain the strength of linear and monotonic relationships between the variables, respectively.

### 2.3 Bootstrap resampling test

The bootstrap method is a fundamental tool to estimate the statistics for a variable of interest [25]. Due to the variation of experimental designs, the interpretation of the parameter relationship is challenged by a large scatter of the data, especially for the (CA + FA + SP) and (CA + FA + SP + RP) datasets. Even if the correlations between two variables turn out to be significant (p < 0.01), the correlation coefficients can still be uncertain in this case. We then checked this by examining the distribution of correlation coefficients with the bootstrap resampling, so that a reliable conclusion can be drawn. Therefore, all obtained correlation coefficients were checked whether they are certain (i.e., within a 95 % confidence interval).

For each dataset, the random sample was taken with replacement to form a paired-bootstrap sample similar in size to the original sample. This process was repeated from 1000 to 10,000 times to produce the paired-bootstrap distribution of correlation coefficients and to construct the 95 % confidence level. This allowed for estimating the statistics for the reported correlation coefficients and also verifying if the trend in which each parameter is correlated or anticorrelated with CS is reliable regardless of limited but diverse experimental data from earlier studies.

### 2.4 Linear regression model

A physically motivated model was adopted to explain the compressive strength using a linear equation in the multivariate form [20,26]. The aim was to compare it with the developed NN model. Since we are interested in the effects of the SP and RP on the CS, only the (CA + FA + SP) and (CA + FA + SP + RP) datasets were investigated in this part. For (CA + FA + SP) and (CA + FA + SP + RP) datasets, the CS was explained using the general formula for the multiple-variable linear regression given by

(1) $$CS={I+\beta }_{1}\left(Cement\right)+\beta_{2}\left(w/b\right)+\beta_{3}\left(CA\right)+\beta_{4}\left(FA\right)+\beta_{5}\left(SP\right)\text{,}$$

(2) $$CS={I+\beta }_{1}\left(Cement\right)+\beta_{2}\left(w/b\right)+\beta_{3}\left(CA\right)+\beta_{4}\left(FA\right)+\beta_{5}\left(SP\right)+\beta_{6}\left(RP\right)\text{,}$$

respectively, as in previous studies [20,26]. I is the variable coefficient and $\beta_{i}$ is the model coefficient associating with the feature i. Note that CS stands for compressive strength (MPa), Cement stands for cement content (kg/m^3^), w/b stands for water-to-binder ratio, CA stands for coarse aggregate content (kg/m^3^), FA stands for fine aggregate content (kg/m^3^), SP stands for superplasticizer dosage (kg/m^3^), and RP stands for recycled plastics content (kg/m^3^).

### 2.5 Neural network algorithm

To implement the neural network, we utilized the Multilayer Perceptron (MLP) regressor algorithm: *sklearn.neural.network.MLPRegressor()* available in *SCIKIT-LEARN* [27]. The MLP regressor can optimize the partial derivatives of the loss function iteratively based on the choices of the activation functions and solvers. The flowchart showing the ML training and testing phase is also presented in Fig. 1. Firstly, the data were divided randomly into the training set (85 %) and test set (15 %). The training data (85 %) were further divided into *K* folds in order to perform the *K*-fold cross-validation. This is an iterative process repeating *K* times and, for each iteration, one partition (validation set) is kept for validating. The remaining *K*-1 parts were used for training the machine. The average performance of the machine can be obtained during the training and validation phase. Here, we employed the *GridSearchCV()* techniques available in *SCIKIT-LEARN* to perform *K*-fold cross validation. To minimize the number of parameters, we also fixed *K* = 5 (i.e., the training data were divided into 5 folds). The test data (15 % of the samples) then were kept unseen so that they were completely new to the machine and were used only for the final evaluation.

A neural network architecture consists of a series of neurons and their connections. For each neuron, x is the input or features representing the set of values that are used by the neuron to predict the output. Within the neuron, each feature is multiplied by a weight, w, that regulates the importance of that feature in predicting the output value, y. Then, the bias, b, is added and the activation function, f, is used to introduce non-linearity to the model, if required. The model for each neuron can then be expressed as

(3) y=f(x⋅w+b),

The neural network is constructed by connecting many neurons. The best neural network can have many layers depending on the nature of the data. Optimization of the neural network can be done by optimizing and finding the weights and biases that provide the smallest average errors.

### 2.6 Hyperparameter tuning and accuracy scores

The model grids were produced to fine-tune the parameters that cannot be directly learned during the training phase. These parameters are referred to as hyperparameters. We selected to fix the number of hidden layers in the neural network to be 1 or 2, and varied the number of neurons in each layer to be between 5 and 50. The grid of neurons was then produced. We investigated four activation functions (f in eq. (3)) including the linear, sigmoid, tanh, and rectified linear unit (relu) functions, all of which are available in the *SCIKIT-LEARN* [27]. To optimize the loss function, we used three different solvers including the standard stochastic gradient descent (SGD), the adaptive algorithm for the first-order gradient-based optimization (Adam) and the Limited-memory Broyden-Fletcher-Goldfarb-Shanno (L-BFGS) algorithms. The learning algorithm can be slightly modified using the parameter α, which controls the penalty on the optimization so that the model generalizes better. We produced the grid of α parameter in the range of 0.001–1000. The grids of these parameters were summarized in Table 1. Fine-tuning these hyperparameters is one of the important steps in training the neural network.

**Table 1:** Number of layers, number of neurons, activation functions, solvers, and regularization parameters investigated in this work.

Hyperparameters	Parameter range/values in model grid
Number of layers	1–2
Number of neurons	5–50
Activation functions	linear, sigmoid, tanh, relu
Solvers	SGD, Adam, L-BFGS
α	0.001–1000

For each grid of the model, the prediction accuracy was evaluated using the coefficient of determination, or *R*-squared:

(4) $$R^{2}=1-\frac{RSS}{TSS}\text{,}$$

where *RSS* and *TSS* are the sum of the squares of residuals and the total sum of the squares, respectively. *R*^*2*^ = 1 means perfect predictions, while *R*^*2*^ < 0 indicates that the prediction by the model is not better than just using the mean of the data.

The uncertainty from the model prediction is also expressed in terms of the mean absolute error (MAE) given by

(5) $$\text{MAE}=\frac{1}{n}\sum_{i=1}^{n}\left|y_{i,pred}-y_{i,true}\right|\text{,}$$

where n is the number of the samples. $y_{i,pred}$ and $y_{i,true}$ are the predicted and the true values of the $i^{th}$ sample. The MAE then provides approximate information on the distance between the true and predicted values. The *R^2^* and MAE from Eqs. (4), (5) are used to evaluate the performance of the LR and NN models.

## 3 Results

### 3.1 Statistical distributions of the data

**Fig. 2:**
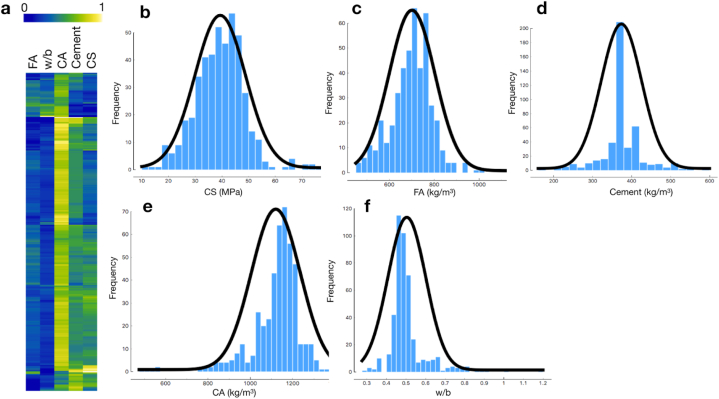
Heat map showing an example of the data distribution of the cement samples in (CA + FA) dataset, where the experimental values of each parameter are scaled to 0–1 (a). The value distribution with the best-fit normal distribution curve is also shown for CS (b), FA (c), Cement (d), CA (e) and w/b (f). See full details of the statistical analysis of all datasets in Table 2.

**Fig. 3:**
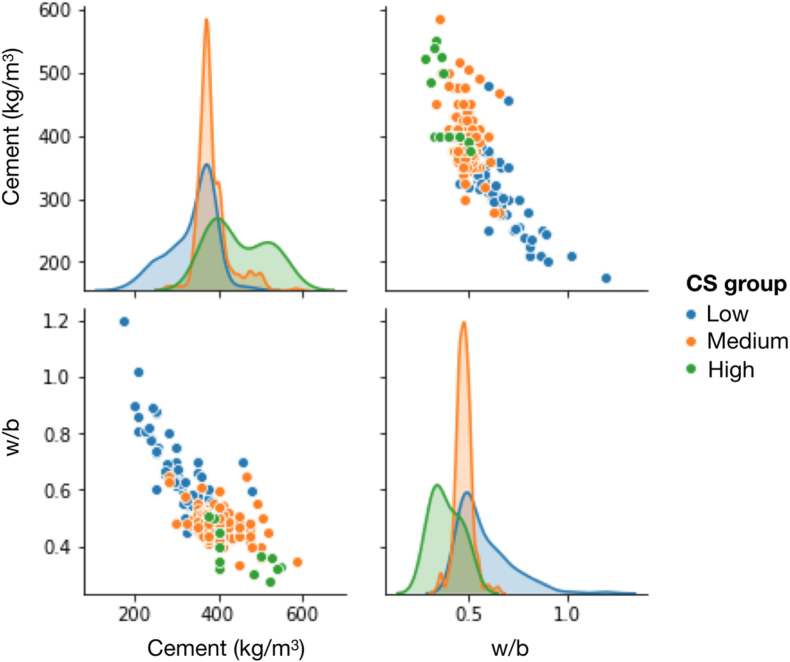
Examples of the pairwise relationships between the Cement and w/b of the cement samples in the (CA + FA) dataset. The compressive strength (CS) was discretized into three groups: low, medium, and high. The diagonal plots represent a univariate distribution of the variables in each column estimated from the KDE. This plot highlighted the strong correlation and anticorrelation between Cement and CS, and w/b and CS, respectively.

The experimental data from each dataset were statistically evaluated before being used to train and test the machine. The variable distribution of the data in the (CA + FA) dataset is shown, as an example, in Fig. 2 (a). The value distribution for each individual parameter with the best-fit normal distribution curve is also presented in Fig. 2 (b)–(f). Note that in our (CA + FA) dataset, only coarse and fine natural aggregates were included as the cementitious composites (without superplasticizer and recycled plastic aggregates). Fig. 3 represents the distribution of the (CA + FA) samples plotted against the Cement and w/b variables when the CS was divided into three groups: low, medium, and high CS. The pattern of associations could be revealed. In fact, we can see that the CS tends to be correlated with Cement, and anticorrelated with w/b.

The statistical values of the model variables for all three datasets are reported in Table 2. These datasets are (CA + FA), (CA + FA + SP), and (CA + FA + SP + RP) datasets, containing 452, 64, and 60 samples, respectively. The highest mean of the cement used was found in the (CA + FA + SP + RP) dataset, while the lowest one was in the (CA + FA) dataset. The CS for both datasets seems to be comparable, however. The samples in the (CA + FA + SP + RP) dataset also have the lowest mean of CA, but a highest mean of FA. The mean of SP in (CA + FA + SP) and (CA + FA + SP + RP) datasets are 6.902 and 6.202 kg/m^3^, which are not much different.

**Table 2:** Statistical values for the model parameters (*Cement, CA, FA, w/b, SP and RP*) of our samples in the (CA + FA), (CA + FA + SP), and (CA + FA + SP + RP) datasets. The Pearson correlation coefficients (*r*_*P*_) and Spearman's rank correlation coefficients (*r*_*S*_) between *CS* and each of the model features are also shown. Note that these coefficients vary between 1 and -1, meaning the perfect correlation and anticorrelation, respectively.

Variables	Mean	Min	Max	Median	Dispersion	*r*_*P*_	*r*_*S*_
(CA + FA) dataset							
*CS* (MPa)	39.358	12.245	75.9	39.541	0.241	–	–
*Cement* (kg/m^3^)	373.655	175	585.366	375	0.139	+0.635 (p « 0.01)	+0.442 (p « 0.01)
*CA* (kg/m^3^)	1120.511	498	1360.976	1141.5	0.103	+0.004 (p = 0.93)	+0.040 (p = 0.393)
*FA* (kg/m^3^)	700.865	451.25	1285	700	0.145	−0.163 (p = 0.0005)	−0.143 (p = 0.0023)
*w/b*	0.504	0.280	1.194	0.48	0.192	−0.634 (p « 0.01)	−0.432 (p « 0.01)
(CA + FA + SP) dataset							
*CS* (MPa)	60.259	25.097	109.831	57.678	0.348	–	–
*Cement* (kg/m^3^)	463.264	312.7	702	453	0.199	+0.760 (p « 0.01)	+0.695 (p « 0.01)
*CA* (kg/m^3^)	1046.256	595	1303	1042	0.170	−0.284 (p = 0.023)	−0.260 (p = 0.038)
*FA* (kg/m^3^)	746.325	568	1166	723.7	0.162	+0.118 (p = 0.354)	+0.043 (p = 0.734)
*w/b*	0.397	0.192	0.661	0.4	0.262	−0.820 (p « 0.01)	−0.826 (p « 0.01)
*SP* (kg/m^3^)	6.902	1.040	35.1	6.450	0.813	+0.325 (p = 0.009)	+0.222 (p = 0.078)
(CA + FA + SP + RP) dataset							
*CS* (MPa)	40.679	17	79.9	37.295	0.423	–	–
*Cement* (kg/m^3^)	500.85	400	615	497	0.1	+0.733 (p « 0.01)	+0.772 (p « 0.01)
*CA* (kg/m^3^)	718.05	300	818	770	0.193	+0.296 (p = 0.022)	+0.216 (p = 0.097)
*FA* (kg/m^3^)	750.535	517	917	777.8	0.144	+0.467 (p = 0.0002)	+0.505 (p « 0.01)
*w/b*	0.371	0.285	0.45	0.36	0.134	−0.781 (p « 0.01)	−0.820 (p « 0.01)
*SP* (kg/m^3^)	6.202	2.5	13.2	6	0.433	+0.491 (p « 0.01)	+0.564 (p « 0.01)
*RP* (kg/m^3^)	45.729	1.11	138	41.9	0.804	−0.036 (p = 0.787)	+0.080 (p = 0.542)

**Fig. 4:**
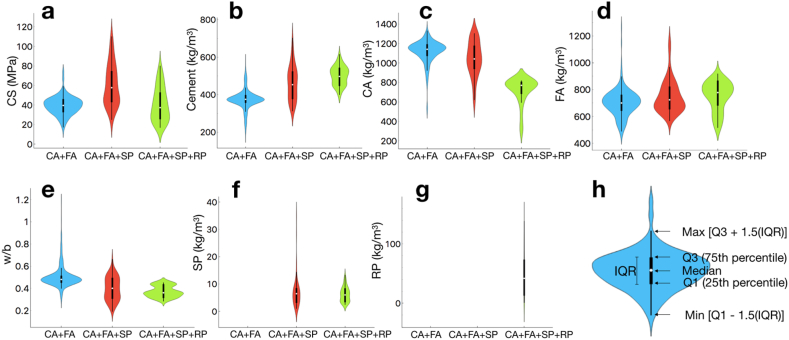
Violin plots showing the distribution of variables including CS (a), Cement (b), CA (c), FA (d), w/b (e), SP (f), and RP (g) comparing between three different datasets: 1) CA + FA (blue), 2) CA + FA + SP (red), and 3) CA + FA + SP + RP (green), with detailed explanation in panel (h). Note that the (CA + FA), (CA + FA + SP), and (CA + FA + SP + RP) datasets contain 452, 64, and 60 samples, respectively.

The violin plots comparing the data distribution among three datasets are also presented in Fig. 4. The mean *CA* among these samples varies between ∼40 and 60 MPa (Fig. 4 (a)). The amount of cement used in the (CA + FA + SP + RP) dataset is significantly larger than that in other datasets, while the CA is significantly smaller (Fig. 4 (b)–(c)). The maximum CA of the (CA + FA + SP + RP) samples is still lower than the mean CA of other datasets. The mean and dispersion of the *FA* and *w/b* also vary across the datasets (Fig. 4 (d)–(e)). On the other hand, the dispersion of RP in our (CA + FA + SP + RP) dataset is quite large (i.e., the violin shape is very thin). The dispersion of the SP in the (CA + FA + SP) dataset is significantly larger than that in the (CA + FA + SP + RP) dataset (i.e., the violin shape is vertically longer as can be seen in Fig. 4 (f)). The dispersion of the RP in the (CA + FA + SP + RP) dataset is also large (Fig. 4(g)). These results emphasized a diversity of the experimental samples fallen into different datasets. Note that the detailed explanation of the violin plot is given in Fig. 4(h).

According to Table 2, the strong correlation and anticorrelation between CS and Cement, and CS and w/b, respectively, were found in all datasets. The FA shows a correlation with the CS only in the (CA + FA) and (CA + FA + SP + RP) datasets. Meanwhile, there is no observed correlation between CS and CA in any datasets, either in a linear or monotonic way (p > 0.01). The SP shows a moderate correlation with CS, whereas the significant correlation between the RP and CS is not observed. This suggested that the RP alone cannot be used to accurately predict CS.

### 3.2 Significance of correlations between CS and model parameters

**Fig. 5:**
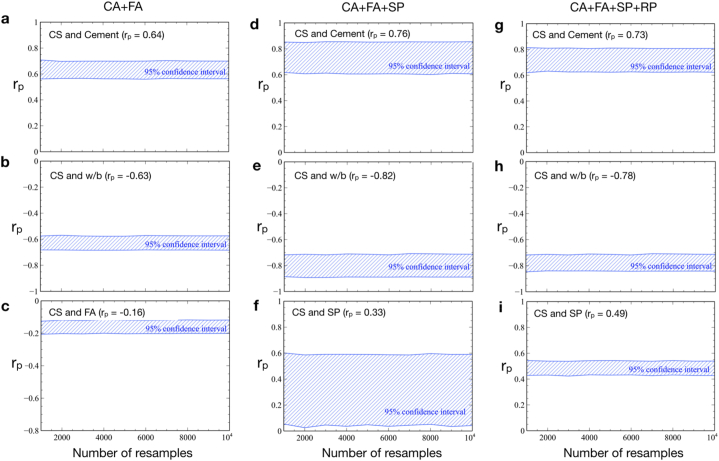
Pearson correlation coefficient values within a 95 % confidence interval inferred using the bootstrap resampling test for the (CA + FA) data (panels a, b, and c), (CA + FA + SP) data (panels d, e, and f), and (CA + FA + SP + RP) data (panels g, h, and i). Only the correlations of the parameters with CS that show *p* < 0.01 are presented.

Due to the limited samples and large scatter of the data, we examined the distribution of correlation coefficients with paired-bootstrap resampling repeated from 1000 to 10,000 times, where a 95 % confidence interval is drawn based on the paired-sample statistics. This is to verify whether the correlation coefficients that show *p* < 0.01 reported in Table 2 are certain. The results are shown in Fig. 5. For the (CA + FA) dataset, it can be seen that 0.57 ≤ *r*_*P*_ ≤ 0.71 for the CS and Cement , −0.67 ≤ *r*_*P*_ ≤ −0.58 for the CS and w/b , and −0.21 ≤ *r*_*P*_ ≤ −0.12 for the CS and FA, with 95 % confidence level (Fig. 5 (a)–(c)). In fact, the *r*_*P*_ for all cases that show *p* < 0.01 and in all datasets seem to be varied approximately within ± 0.1 of the reported values in Table 2 (Fig. 5 (a)–(e) and Fig. 5 (g)-(i)), except the *r*_*P*_ of CS and SP in the (CA + FA + SP) case that seems to be uncertain (Fig. 5 (f)). The obtained correlations here were used for the guideline of downstream NN modelling and later discussion.

### 3.3 Linear regression (LR) models and accuracy

The compressive strength was explained using the linear equations in the multivariate forms given in Eqs. (1), (2). The best-fit LR equations are expressed in Table 3. For (CA + FA + SP) dataset, the prediction accuracy of the LR model is 0.564 and the MAE is 11.149. On the other hand, for the (CA + FA + SP + RP) dataset, the LR model can adequately explain the data, with the accuracy of 0.721, and the acceptable MAE of 6.717. Despite the diversity of data due to different experimental designs, all obtained CS equations still show similar trend that the Cement, CA, and FA terms have positive signs while the w/b, SP and RP terms are negative. In other words, all LR models suggested that, for the majority of the data, increasing Cement, CA or FA should result in increasing CS, whereas increasing w/b, SP or RP decreases CS.

**Table 3:** Best-fit results from the linear regression (LR) models.

Dataset	CS equations	*R*^*2*^	MAE
CA + FA + SP	CS=20.296+0.069(Cement)−126.531(w/b) +0.023(CA)+0.047(FA)−0.184(SP)	0.564	11.149
CA + FA + SP + RP	CS=95.755+0.016(Cement)−267.115(w/b)+0.025(CA)+0.034(FA)−0.379(SP) −0.112(RP)	0.721	6.717

### 3.4 Neural network models and accuracy

The best CS forecasting NN models are presented in Table 4. Note that the number of neurons, activation functions, solvers, and α were fine-tuned via the *K*-fold cross-validation technique using *GridSearchCV()*, where *K* was fixed at 5. For both (CA + FA + SP) and (CA + FA + SP + RP) datasets, the best-fit models required the relu function, with the L-BFGS solver. The obtained accuracy was quite high, especially in the case of the (CA + FA + SP + RP) sample that showed *R*^2^ = 0.914 and MAE = 2.336. The *R*^2^ scores obtained from the test set were slightly smaller than those obtained from the training/validation set, indicating that our NN models did not overfit the data (i.e., the model performances were still good when evaluated using the test data that were completely new to the machine).

**Table 4:** Obtained parameters and prediction accuracy from the neural network (NN) models.

Dataset	Neurons	Activation function	Solver	α	*R*^2^ train [test]	MAE train [test]
CA + FA + SP	15,15	relu	L-BFGS	11.288	0.934 [0.892]	1.911 [4.328]
CA + FA + SP + RP	25,25	relu	L-BFGS	8.857	0.938 [0.914]	1.153 [2.336]

**Fig. 6:**
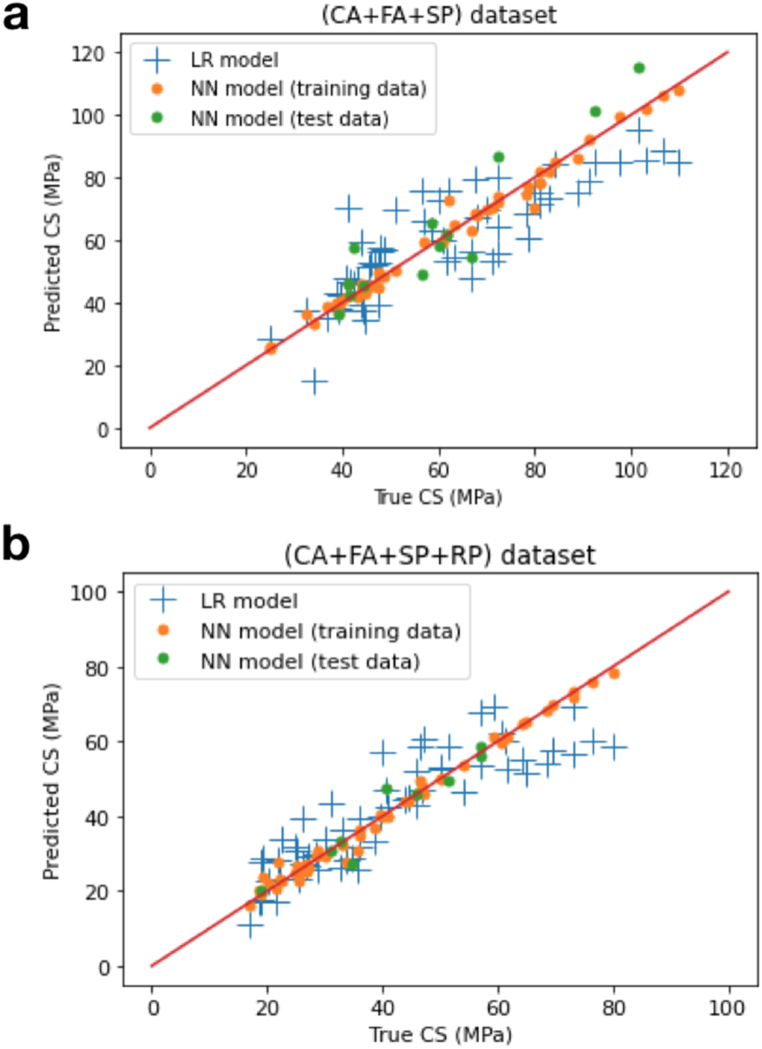
Comparison between the prediction results of the LR and NN models for the different datasets: (CA + FA + SP) (a), and (CA + FA + SP + RP) (b). The red solid lines represent the perfect prediction lines.

The scatter plots of all actual and predicted values of the CS compared between the NN and LR models are presented in Fig. 6 (a) and (b) for the (CA + FA + SP) and (CA + FA + SP + RP) datasets, respectively. We also drew the line representing the perfect prediction in the plot. The LR model seemed to significantly underpredict the high CS data for all datasets. In all cases, the accuracy of the NN model was significantly higher than the LR model, while the MAE was significantly smaller. This clearly illustrated the potential use of the NN models to make an accurate prediction of CS.

### 3.5 Dependence of CS on the model parameters

**Fig. 7:**
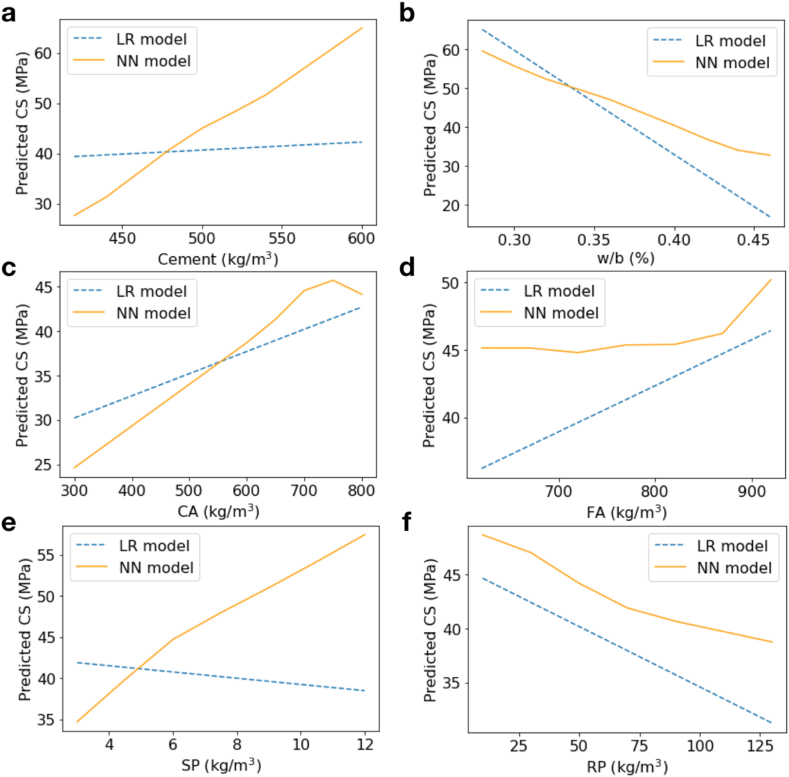
CS variations with Cement (a), w/b (b), CA (c), FA (d), SP (e), and RP (f) as predicted by the LR (blue-dashed lines) and NN (orange-solid lines) models. When varying each of these parameters, the rest parameters were fixed at their mean values from the (CA + FA + SP + RP) dataset.

Now, we investigated how the CS changes with each of the NN model features, compared to the LR cases. To illustrate this, we selected to use the models obtained from the (CA + FA + SP + RP) dataset, since both LR and NN models displayed the best *R*^*2*^ and MAE. We varied the model features one by one and observed how the CS changed according to that feature. Other parameters, except the one that was varying, were fixed at their mean values. The results are presented in Fig. 7. For the LR model, an increasing trend of CS with Cement, CA, and FA, with the slope of the profile controlled by the parameter coefficients (as in Table 3) was observed. Based on the LR equation, the CS also linearly decreased with increasing w/b, SP, and RP (Fig. 7 (b), (e) and (f), respectively). Contrarily, for the NN model, the trends of altering CS with these features were non-linear. To better explain the data, the CS needed to be more sensitive with Cement and CA than what was suggested by the traditional LR model (Fig. 7 (a) and (c), respectively). The NN model suggests that the dependence of CS on FA is less clear than what proposed by the LR model (Fig. 7 (d)). Moreover, the NN model revealed that the CS should increase with SP, rather than decrease as suggested by the LR model (Fig. 7 (e)). These results highlighted significant differences in how the NN and LR models explain the data. It should be noted that the NN model provided significantly higher *R*^2^, so the trend of orange lines in Fig. 7 should provide an accurate reflection of the true nature of parameter associations among the general cement samples extensively gathered from earlier studies.

## 4 Discussion

Firstly, our data samples were statistically evaluated by dividing them into three groups. The Pearson and Spearman's correlations between the 28-day CS and each of the model parameters for each dataset were calculated individually. Spearman's correlation uses rank statistics instead of the actual numeric values to evaluate the monotonic relationships. When | *r*_*S*_ | > | *r*_*P*_ |, the variables changed with CS in the same or opposite direction but not likely at a constant rate. In some cases, we found that | *r*_*P*_ | > | *r*_*S*_ |, which was probably driven by the samples in the tails of the distribution that had a high influence relative to their ranked values. However, there was no significantly large gap between the Pearson and Spearman's correlations for each pair of variables.

Undoubtedly, CS showed strong correlation and anticorrelation with Cement and w/b, respectively, for all datasets. This agrees with previous literature such as Hossain et al. [28]. On the other hand, it was suggested that RP aggregate content substantially impacts the CS of cementitious composites mixed with RP aggregates [20]. Here we found no significant correlation between CS and RP, either in linear or non-linear ways. This difference might depend on the choices of how the data were selected and separated into the dataset. Nevertheless, we found that SP also had a substantial impact on the compressive strength of cementitious composites (i.e., its correlation with CS was moderate), in agreement with previous literature such as Mohammed et al. [13].

Based on the bootstrap resampling tests, the *r*_*P*_ between CS and SP was certain only in the cementitious composites incorporated RP aggregates (Fig. 5 (i)). Regarding the deviation of samples among different datasets, the trends in which each parameter correlated or anticorrelated with CS were quite certain, however. Most of the correlation coefficients with *p* « 0.01 seemed to vary only within ± 0.1 (95 % confidence level) of the reported values. Since the majority of correlations were certain, accumulating more new data might not change the obtained correlations here. This was also probably the reason why the common trends of the linear relationships of the parameters among three different datasets could still be implied using the LR models (Table 3). Note that, while the LR models adequately predicted CS in all datasets (*R*^*2*^∼ 0.45–0.72), they tended to overpredict and underpredict the data for low and high CS, respectively. Interestingly, similar misprediction trends at the low and high end of CS were also found in nonlinear multivariable regression analysis in the natural aggregate samples [15]. This means that the traditional regression model, whether it is linear or non-linear multivariable, cannot provide an accurate prediction of CS.

The generic LR equations obtained here were consistent in the way that an increase of CS for all datasets required an increase of Cement, CA and FA, but required a decrease in RP. This agreed with the previous LR analysis on the self-compacting concrete samples containing recycled plastics, carried out by Faraj et al. [20]. However, our trend of increasing CS with decreasing w/b and SP contradicted their results. Note that Faraj et al. [20] considered various curing periods simultaneously, not only 28-day curing time, so the associations of CS with other parameters might not be easily disentangled in a linear way.

Previous studies revealed that the *CS* could be accurately retrieved using the NN technique [[16], [17], [18], [19], [20]]. There were differences among the NN models used in these studies. For example, Faraj et al. [20] used a feedforward NN while Bu et al. [19] used the multilayer perceptron, which is a special case of the feedforward NN. In the multilayer perceptron algorithm, every layer is fully connected so it can efficiently perform feature learning. This work then contributes with one more independent analysis on the potential use of the multilayer perceptron for estimating unconfined CS. Contrarily, some studies suggested that the NN model could not outperform the traditional non-linear approach (e.g., Mohammed et al. [29]). The inconsistencies might arise due to different choices of data selection, the nature of the materials, and errors in the experimental data employed in different studies. Instead of focusing on a specific experimental setting as in previous works, the model developed here is likely mixture-type independent that can predict the CS regardless of the experimental designs. The fact that our NN model performed well and could also adapt to the new data suggests that it could be widely applicable in the future by, e.g., saving considerable cost and time for cement preparation with specific CS of interest in both laboratory and industrial scales.

All NN models preferred the L-BFGS solver. This is probably because the standard solver such as the adam algorithm is more suitable for large datasets (e.g., more than thousands of samples), whereas the L-BFGS solver can perform better and converge faster on relatively small datasets [27]. Although there were several explanatory variables for CS, the plots in Fig. 7 showed how the CS varies solely by each specific feature. Both LR and NN models showed a similar trend of increasing CS with increasing Cement and CA, and with decreasing w/b. When compared between both models, each of these variables had different influential sensitivity to CS. Our NN model suggested that an increase of 100 kg/m^3^ of CA could increase CS by ∼4 MPa, while the use of CA of more than 700 kg/m^3^ as a mixture could decrease the CS. Note that CA and FA are mixtures that have higher CS than cement, so with the suitable dosage of both types of aggregates the CS can increase. Nevertheless, how the CS varies with FA is less clear and nonlinear (Fig. 7 (d)). This is because the RP was normally used to replace the FA, so the amount of FA may be altered by the RP, hence the trend of the CS varying with FA is less clear compared to the case of CA.

From Fig. 7 (e), the LR and NN models predicted inconsistent trends of the dependence of CS on SP. The use of superplasticizer within an optimal limit can boost the compressive strength of hardened concrete by improving concrete compaction efficiency, hence enhancing the CS (e.g, Mohammed et al. [13], Needhidasan et al. [30], Dash et al. [31], Tannous et al. [32]). Therefore, the LR models preferred the framework where the used SP dosage was beyond the optimum limit and the CS was reduced with SP. This was, however, ruled out by the NN model that supported an increasing trend of CS with SP, which was preferable due to the higher accuracy score. The SP usages for most of the cement samples investigated here then should be within the optimal limit that still helps promote the CS [33]. In fact, the optimal applied dosage of SP is 1–2% per unit weight of cement, which is the range of SP used in all literature included in this work. It was also well consistent with the positive correlation coefficients found between CS and SP. An additional 1 kg/m^3^ of SP could enhance the CS by ∼2 MPa.

Contrarily, by adding 1 kg/m^3^ of RP as the mixture, the CS could reduce by ∼0.2 MPa. The aforementioned trends agreed with experimental results in some previous literature. For example, Kaewpikul et al. [10] found that an increased polyvinyl chloride (PVC) waste caused a reduction in CS of PVC-incorporating cement. Aocharoen & Chotickai [34] used high-density polyethylene (HDPE) sand as fine aggregates and found that a decrease in the average compressive strength with increasing HDPE sand was likely explained by a quadratic equation, rather than a linear relationship. This trend was well justified by our NN model where the prediction profiles of the CS showed a negative, decreasing slope with increasing RP (Fig. 7 (f)).

Note that other factors affecting the compressive strength of the materials such as the shape of plastic aggregates [35,36] and the incorporation of the calcifying bacteria in the biocement experiments [[37], [38], [39], [40], [41]] were not considered here yet. While recent studies found that the well-established models could not easily explain the cementitious composites incorporating plastic aggregates due to the different characteristic nature of various aggregates [9,15,34], our developed NN model could accurately forecast the CS without the need to determine the size and shape of plastic aggregates (*R*^*2*^ > 0.9 for (CA + FA + SP + RP) samples). Even though there might be some biases due to the data selection and the past experiments were historically conducted for different reasons, the collected data so far should be representative enough of the underlying populations at least in the way that we can draw meaningful information between the cement parameters.

The developed NN models are general enough that they do not require the discrimination of the characteristics of RP and other mixtures in advance. They are also general for many types of cement-based materials included in the datasets, for example, self-compacting concrete, mortar, waste-incorporating cement composites, etc. It should be noted that the increase of CA in relation to FA can be expressed in terms of the FA-to-CA ratio either by mass or volume. The increase of FA content will increase compressive strength only when the FA-to-CA ratio is much higher than the ACI 544.3R-2008 standard ratio of 0.6 [42], which is not always the case for the data in this work. The SP quantity can also be expressed as a ratio, e.g., per unit weight of cement. However, we would like to highlight the influence of each parameter on the CS individually, so such a ratio was not yet taken into consideration in our work. Extrapolating the model far beyond the minimum and maximum values of each parameter or carrying out a reliable multivariate-data test, e.g., a 2-sample test for 2D variables, is still non-trivial due to the limited size of our collected samples. More new, updated data will be required to quantitatively evaluate the impact due to the variation of several parameters simultaneously.

## 5 Conclusion

The NN holds promise for large-scale analysis of data collected from various experimental setups. The global dependency of CS on each cement mix can be revealed, without specifying the secondary factors such as the setting time, the type of SP and the structure of RP in advance. It outperformed the conventional LR approach in all cases. The NN model predicted that a 100 kg/m^3^ increase in CA might promote the CS by ∼4 MPa while using a mixture of more than 700 kg/m^3^ of CA could lower the CS. Despite the uncertain correlation between CS and SP, the CS might be increased by 2 MPa by adding 1 kg/m^3^ of SP while being within the optimal limit. On the other hand, an increase of 1 kg/m^3^ in RP would cause a 0.2 MPa reduction in CS.

The prediction trends governed all majority types of binders or mixtures widely used in previous literature. Our mixture-type independent models can assist in the manufacture of cement with a particular CS of interest on both laboratory and industrial scales. Considering the influence of other factors or including more features is possible, but this can lead to more model degeneracy and the NN may need to be evaluated, or re-trained, on a case-by-case basis. Finally, while the multilayer perceptron shows good applicability in this work, other NN models such as ANN-GA [43] and ANN-GEP [44] are also worth investigating in the future. Using new ML methods such as XGBoost, CatBoost, LightGBM or hybrid models may further improve the model accuracy, which is the subject of future study.

## Data availability statement

The data used in this study are provided in the supplementary files (or available in https://github.com/PChainakun/Data_CS_NN).

## CRediT authorship contribution statement

**Jindarat Ekprasert:** Conceptualization, Data curation, Investigation, Resources, Supervision, Writing – original draft, Writing – review & editing. **Natthagrittha Nakhonthong:** Formal analysis, Investigation, Methodology, Validation. **Vanchai Sata:** Formal analysis, Supervision, Validation. **Poemwai Chainakun:** Formal analysis, Investigation, Methodology, Software, Validation, Visualization, Writing – original draft, Writing – review & editing.

## Declaration of competing interest

The authors declare that they have no known competing financial interests or personal relationships that could have appeared to influence the work reported in this paper.

## Figures and Tables

**Table A1:** Comprehensive literature where the data were collected and used in this work.

Authors	Detailed Samples
Kumar & Baskar 2015	In this study, E-waste plastics were used as a replacement for coarse aggregate, which was used in the form of chips and flaky material and was obtained solely from computer and accessory equipment. When mixing and curing the concrete specimens, they also used potable water that is free of impurities and salt. The concrete mixtures and further information provided: 1) E-plastic 2) Water/cement ratio 3) Cement 4) Water 5) Fine aggregate 6) Coarse aggregate
Abdelatif et al., 2018	Aggregate characteristics including aggregate type, maximum size, percentage passing sieve, and silt/clay/dust contents are also included in the data. The information includes mix design ratios for cement content, water, fine and coarse aggregate, as well as any additional admixtures. In this study, the mix design data, which included more than 1000 mix designs and more than 1400 fine and coarse aggregate test results, were categorized using concrete grades 25 and 30. The concrete mixtures and further information provided: 1. Type and content of fine and coarse aggregates 2. Maximum size of coarse aggregates 3. Percentage of aggregates passing sieve 0.6 mm 4. Cement content (OPC) 5. Water/cement ratio 6. Additive type and dosage if used
Gravina et al., 2021	A large variety of plastic aggregate types were covered by the experimental database of 370 concrete mix designs compiled from 60 studies in this sample. Curing conditions include using either water curing or a standard fog room environment. Variety of plastics used in concrete mixtures: 1. LDPE-Coarse 2. LDPE-Fine 3. PET-Coarse 4. PET-Fine 5. E-Waste-Coarse 6. PVC-Coarse 7. PVC-Fine 8. PP-Coarse 9. PP-Fine 10. HDPE-Coarse 11. HDPE-Fine 12. PC-Fine
Faraj et al., 2022	Recycled plastic aggregates were used in sustainable self-compacting concrete (SCC) mixes. In addition, 400 test results from a large amount of experimental data that varied RP aggregate concentrations, w/b ratios, and curing regimes were examined. The concrete mixtures and further information provided: 1. Recycled plastic 2. Binder 3. Water/binder ratio 4. Curing time (days) 5. Superplasticizer 6. Fine aggregate 7. Coarse aggregate

## Appendix A More details of the experimental data

To emphasize the variation of experimental designs for the data employed in this work, the comprehensive literature where the data were collected is summarized in Table A1. Note that some authors also employed the data from previous studies.

## Appendix B Nomenclature

$\beta_{i}$: Model coefficient associating with the feature i
b: Bias in the neural network model
CA: Coarse aggregate
CS: Compressive strength
E-waste: Electronic waste
f: Activation function in the neural network model
FA: Fine aggregate
HDPE: High Density Polyethylene
I: Variable coefficient in the linear regression model
KDE: Kernel density estimate
LDPE: Low Density Polyethylene
LR: Linear regression
MAE: Mean absolute error
ML: Machine learning
MLP: Multilayer perceptron
MPa: Megapascal
n: Number of the samples
NN: Neural network
OPC: Ordinary Portland cement
PA: Plastic aggregate
PAC: Plastic aggregate concrete
PC: Polycarbonate
PET: Polyethylene terephthalate
PP: Polypropylene
PVC: Polyvinyl chloride
*r*_*P*_: Pearson correlation coefficients
*r*_*S*_: Spearman's rank correlation coefficients
RP: Recycled plastic
RSS: Sum of the squares of the residual
SCC: Self-compacting concrete
SP: Superplasticizer
TSS: Total sum of the squares
w/b: Water-to-binder ratio
w/c: Water-to-cement ratio
w: Weight in the neural network model
x: Input in the neural network model
y: Output in the neural network model
$y_{i,pred}$: Predicted values of the $i^{th}$ sample
$y_{i,true}$: True values of the $i^{th}$ sample
